# Low interleukin-1**α** messenger RNA levels predict decreased overall survival time of patients with urinary bladder carcinoma

**DOI:** 10.1054/bjoc.2000.1584

**Published:** 2001-02

**Authors:** M Seddighzadeh, G Steineck, O Jansson, P Larsson, H Wijkström, J Adolfsson, N Portwood, J Hansson, S Linder

**Affiliations:** Radiumhemmet's Research Laboratory, Cancer Center Karolinska, Karolinska Institute and Hospital, S-171 76 Stockholm; Clinical Epidemiology; Department of Oncology-Pathology, Karolinska Institute and Hospital, S-171 76 Stockholm;; Section of Urology, Department of Surgical Sciences, Karolinska Institute and Hospital, Stockholm, S-171 76, Sweden; Department of Urology, C1, Stockholm South Hospital, Stockholm, S-118 83; Division of Urology, Department of Surgery, Anesthesiology, Radiology and Orthopedic Surgery, Karolinska Institute, Huddinge Hospital, Huddinge, S-141 86, Sweden

**Keywords:** urinary bladder cancer, interleukin-1, urokinase

## Abstract

Due to our inability to exactly characterize tumours, many patients with urinary bladder cancer undergo unnecessary surgery or cytostatic therapy. We have here studied the expression of the cytokine interleukin-1α (IL-1α) in 73 human bladder carcinomas in relation to patient survival, and examined possible relationships between IL-1α and urokinase plasminogen activator (uPA) expression. Expression levels of IL-1α and uPA mRNA were determined by RT-PCR using the quantitative TaqMan technique. The levels of IL-1α mRNA expression did not differ significantly between tumours of different grade or stage. Calculation of the overall survival rates showed a decreased overall survival time for patients with low levels of IL-1α mRNA in their tumours (log rank; *P* = 0.0002, median follow up: 37 months). Low tumoral IL-1α expression predicted decreased survival of patients with poorly differentiated tumours (*P*< 0.005) and of patients with invasive tumours (*P* = 0.02). uPA expression was about 4-fold increased in poorly differentiated tumours. High levels of uPA mRNA were associated with decreased overall survival times (log rank; *P* = 0.032, *n* = 60). We conclude that IL-1α is important for bladder cancer biology, and that measurements of this cytokine may be useful in pre-treatment characterization of urinary bladder cancer. © 2001 Cancer Research Campaign http://www.bjcancer.com


British Journal of Cancer (2001) 84(3), 329–334
© 2001 Cancer Research Campaign
doi: 10.1054/ bjoc.2000.1584, available online at http://www.idealibrary.com on IDEAL

http://www.bjcancer.com

### Low interleukin-1α messenger RNA levels predict decreased overall survival time of patients with urinary bladder carcinoma

**M Seddighzadeh[1], G Steineck[2], O Jansson[3], P Larsson[4], H Wijkström[5], J Adolfsson[5], N Portwood[1], J Hansson[1] and S Linder[1]**

[1]Radiumhemmet's Research Laboratory, Cancer Center Karolinska; [2]Clinical Epidemiology; Department of Oncology-Pathology; [3]Section of Urology, Department of Surgical Sciences, Karolinska Institute and Hospital, S-171 76 Stockholm; [4]Department of Urology, C1, Stockholm South Hospital, S-118 83 Stockholm; [5]Division of Urology, Department of Surgery, Anesthesiology, Radiology and Orthopedic Surgery, Karolinska Institute, Huddinge Hospital, S-141 86 Huddinge, Sweden

**Summary** Due to our inability to exactly characterize tumours, many patients with urinary bladder cancer undergo unnecessary surgery or cytostatic therapy. We have here studied the expression of the cytokine interleukin-1α (IL-1α ) in 73 human bladder carcinomas in relation to patient survival, and examined possible relationships between IL-1α and urokinase plasminogen activator (uPA) expression. Expression levels of IL-1α and uPA mRNA were determined by RT-PCR using the quantitative TaqMan technique. The levels of IL-1α mRNA expression did not differ significantly between tumours of different grade or stage. Calculation of the overall survival rates showed a decreased overall survival time for patients with low levels of IL-1α mRNA in their tumours (log rank; $P = 0.0002$, median follow up: 37 months). Low tumoral IL-1α expression predicted decreased survival of patients with poorly differentiated tumours ($P < 0.005$) and of patients with invasive tumours ($P = 0.02$). uPA expression was about 4-fold increased in poorly differentiated tumours. High levels of uPA mRNA were associated with decreased overall survival times (log rank; $P = 0.032$, $n = 60$). We conclude that IL-1α is important for bladder cancer biology, and that measurements of this cytokine may be useful in pre-treatment characterization of urinary bladder cancer. © 2001 Cancer Research Campaign http://www.bjcancer.com

**Keywords**: urinary bladder cancer; interleukin-1; urokinase

Our inability to forecast the natural, untreated, history of invasive urinary bladder cancer makes patients with the disease undergo unnecessary surgery or cytostatic therapy. To prevent high-grade tumours with submucosal growth (T1G3) from progressing to invade the muscle layer, the whole urinary bladder may be removed (radical cystectomy). However for some patients more conservative means would suffice (Steineck et al, 1997). To prevent possible micrometastases from growing to lethal macrometastases cytostatic drugs are given as a precaution (neoadjuvant or adjuvant) to patients with muscle invasive disease. About half, depending on patient selection for the operation, of these patients do not have micrometastases and would be cured by cystectomy only (Hellsten and Steineck, 1997). In the choice between the possibility of unnecessary side effects and of unnecessary premature death most subjects wish to optimize the chance of survival (Heningsohn et al, submitted). We need a better tumour characterization to diminish the number of patients being overtreated.

Death in urinary bladder cancer is often preceded by a continuous tumour growth into the muscle layer and metastatic spread to retroperitoneal lymph glands, visceral organs, skeleton or the brain. Metastatic cancer cells acquire the ability to degrade the extracellular matrix (Liotta and Stetler-Stevenson, 1993), and it is likely that proteases are important for the ability of malignant urothelial cells to penetrate biological barriers such as capsules or vessel walls. High levels of urokinase plasminogen activator (uPA) in tumour extracts have been associated with poor long-term survival in urinary bladder cancer (Hasui et al, 1992). Many proteases, including uPA (Pyke et al, 1991), various metalloproteases (Wolf et al, 1993; Engel et al, 1994; Okada et al, 1995) and cathepsin D (Tetu et al, 1993) are not primarily expressed by tumour cells, but rather by fibroblasts in the tumour stroma. The mechanisms that induce protease expression in stromal fibroblasts are not well understood. FGF-2 (fibroblast growth factor-2) immunostaining correlates to uPA and stromelysin-3 expression in breast cancer (Visscher et al, 1995; Linder et al, 1998). In vitro studies have demonstrated that FGF-1 and FGF-2, EGF, PDGF-BB and IFN-γ and IL-1 are capable of inducing uPA expression in human fibroblasts (Michel and Quertermous, 1989; Bhat-Nakshatri et al, 1998; Sieuwerts et al, 1999).

IL-1 is considered to be the major pro-inflammatory cytokine (Dinarello, 1996). IL-1α is expressed in two forms, IL-1β and IL-1α, which in most studies show indistinguishable biological activities (Dinarello, 1997). A number of different effects of IL-1 on tumours and on tumour cell lines have been reported. IL-1 has growth inhibitory and cytocidal effects on tumour cell lines (Onozaki et al, 1985). IL-1 upregulates host defences and functions as an immunostimulatory agent (Dinarello, 1996) and has also been reported to have anti-angiogenic activities (Norioka et al, 1987). IL-1 shows some antitumoral activity in melanoma and osteosarcoma patients (Janik et al, 1996) and has been used

*Received 19 June 2000*
*Revised 18 September 2000*
*Accepted 18 October 2000*

*Correspondence to:* Stig Linder in clinical trial (Worth et al, 1997). In contrast to these anti-tumoral effects, IL-1 may promote metastasis of some tumours by stimulating tumour cell-endothelial cell adhesion (Burrows et al, 1991). In addition, IL-1 enhances the expression of matrix degrading enzymes in fibroblasts, including uPA (Leizer et al, 1987; Michel and Quertermous, 1989) and stromelysins (Brinckerhoff et al, 1992). Conditioned media from some breast cancer cell lines induce uPA expression in fibroblasts, and this induction has been shown to be mediated by IL-1α (Bhat-Nakshatri et al, 1998).

We have scant data for the role of endogeneous IL-1 in human malignancy. IL-1β has been reported to be expressed by most breast carcinomas, and high IL-1β content is often associated with tumour invasiveness (Jin et al, 1997). IL-1 activity has also been detected in human bladder cancer cell lines (Hayashi et al, 1994). Presence of inflammation has been implicated as a determinant of better prognosis of bladder tumours (Flamm, 1990). The degree of inflammation induced by Bacillus Calmette-Guerin (BCG) treatment of superficial bladder carcinomas probably correlates with the anti-tumour response (Bohle, 1999). Interleukin is one of many cytokines released during an inflammatory process. We here report on analyses of IL-1α mRNA expression in urinary bladder tumours using TaqMan PCR.

### SUBJECTS AND METHODS

### Patient cohort

Fresh tumour biopsies from a consecutive population-based series of more than 600 newly diagnosed cases of bladder cancer tumours were collected in Stockholm during 1995–1996. Patients with muscle-invasive tumours (T2+) underwent a radical cystectomy, external beam radiotherapy or transurethral resection only and sometimes cytostatic chemotherapy was also given. Patients with tumours confined to the mucosa were predominately treated with transurethral resection, sometimes with addition of intravesical instillations of BCG or cytostatic drugs. For the present study 95 of these patients were chosen at random, stratified into two equal-sized groups (G1 + G2a and G2b + G3). Before transurethral resection, 4 tissue samples were collected from each tumour by cold-cup biopsy and immediately frozen at −80˚C. One of the frozen tissue samples was used for extraction of RNA and another was cut into 5 µm thick sections. The first and the last section were stained and examined histologically to evaluate tumour content. Based on histopathological examination of material from the diagnostic transurethral resection, we defined Ta tumours as only growing in the mucosa, T1 tumours as invading the submucosa (lamina propria) only and T2+ tumours as invading the main muscle layer (Hall and Prout, 1990). The system of grading follows the tradition in Stockholm started during the 1960s (Bergkvist et al, 1965), distinguishing grade 2a from 2b tumours. It is a modification of the WHO system and concordant with the recent suggestion from an international panel of pathologists to separate low and high malignant urinary bladder tumours (Epstein, 1998). In some of the analyses, we group grade 2b and grade 3 tumours together.

Death of any cause was used as the main outcome variable over the relatively short period of follow-up, to avoid conceptual and practical difficulties in defining death cause (see Shipley et al, 1998; Stein et al, 1998).

### RNA extraction and RT-PCR

RNA was extracted from tumours using Trizol (Life Technologies). DNase treated RNA (0.5 µg) was reverse transcribed to cDNA using 200 U Superscript reverse transcriptase, 50 mM Tris-HCl pH 8.3, 75 mM KCl, 3 mM $MgCl_2$, 0.1 M dithiotreitol, 10 mM dNTPs and 100 ng of $Pd(N)_6$ random hexamer primers (Life Technologies).

PCR amplifications were performed at a final concentration of 1 × PCR buffer, 400 µM dNTP, 6 mM of $MgCl_2$ and 1.25 U of Taq Gold polymerase (all from PE Biosystems) in a total volume of 25 µl. Primer and probe concentrations were as follows: u-PA: 50 nM of sense primer, 300 nM antisense primer (Life Technologies) and 50 nM probe (PE Biosystems); 18S rRNA: 200 nM sense, 80 nM antisense primer and 100 nM probe (PE Biosystems).

Amplification of IL-1α and 18S rRNA (endogenous control) cDNA templates were performed in multiplex form using a kit from PE Biosystems. Amplification of uPA and 18S rRNA cDNA templates were performed in singleplex form. An ABI-prism 7700 Sequence Detector (PE Biosystems) was used for TaqMan PCR. A cDNA equivalent of 25 ng RNA was amplified in triplicate. The following conditions were used: 94˚C for 10 min to activate the Taq Gold polymerase and then denaturation at 94˚C for 15 s, primer and probe annealing and extension at 62˚C for 1 min in a total of 40 cycles.

### Primers and probes

Primers and probes were obtained from PE Biosystems or Life Technologies and designed using the Primer express software (PE Biosystems). u-PA forward primer: 5′-GGG AAA CAT AAT TAC TGC AG3′ (nucleotide 2742–2760 and 2954–2958 in the human u-PA gene sequence); reverse primer: 5′-AAG CGG CTT TAG GOC CAC-3′ (nucleotide 2994–3011); probe: 5′-TGC ACA TAG CAC CAG GGT CGC CT′-3′ (nucleotide 2970–2992). 18S rRNA: forward primer: 5′-CGG CTA CCA CAT CCA AGG AA-3′ (nucleotide 551–570); reverse primer: 5-GCT GGA ATT ACC GCG GCT-3′ (nucleotide 720–737); probe: 5′-TGC TGG CAC CAG ACT TGC CCT C-3′ (nucleotide 699–720).

### Fluorescence measurements, calculations and validation

TaqMan PCR is based on the use of a dually labelled probe that is consumed by the 5′-3′ nuclease activity of Taq-polymerase, resulting in accumulation of fluorescence during each PCR cycle (Heid et al, 1996). The Ct (threshold cycle) value is defined as the PCR cycle where the fluorescent signal increases above a fixed threshold. In this study, amplification of IL-1α or uPA sequences was normalized to 18S rRNA amplification, resulting in a △Ct value. The △Ct of the sample was related to the △Ct of a internal standard cDNA that was included in all amplification runs (by subtracting internal standard △Ct from sample △Ct, resulting in △△Ct values). This standard was obtained from MDA-MB-231 breast cancer cells, known to express both IL-1α and uPA. Finally, △△Ct values were converted to arbitrary units (a.u.) by calculating the $2^{-\triangle\triangle ct}$ value.

When insufficient amounts of cDNA were used, the Ct values for IL-1α and uPA resulted in unacceptable variations in the triplicate values. Based on a series of preliminary experiments, we decided

© 2001 Cancer Research Campaign to only evaluate samples resulting in Ct values of 18S rRNA amplification of ≤ 20 (for IL-1α) and ≤ 23 (for uPA). Different cut-offs were used since the mean Ct values of IL-1α amplification were higher than those of uPA amplification. Using these cut-offs reduced the number of clinical samples which could be evaluated by approximately one third.

The validity of the RT-PCR method for determination of IL-1α and uPA expression was evaluated using cDNA from the MDA-MB-231 breast cancer cell line. In 8 independent amplifications, IL-1α: 18S rRNA and uPA:18S rRNA expression showed standard deviations of 25% and 15%, respectively.

### Statistical analysis

Overall survival of patients was calculated by the Kaplan- Meier method (Kaplan and Meier, 1955). The median values were used as cut-offs for discriminating between patients with high vs. low IL-1α (0.040 a.u.) respective uPA levels (0.084 a.u.). To analyse differences between the groups the logrank test was used. The unpaired Student's *t*-test and the Chi-square test were used for comparison of IL-1α mRNA levels in bladder cancer tissues according to tumour grade or stage of the primary tumour. After dicotomization of the independent and dependent variables, we calculated the relative risks as the ratio of proportions, e.g. as the percentage of deaths among patients with a high IL-1α expression divided by the percentage of deaths among patients with a low IL-1α expression. The 95% confidence interval of the relative risk was calculated with the Mantel-Haenszel method (Rothman and Greenland, 1998). To compare survival in different groups, adjusted for potential confounding factors, different Cox proportional hazard models were calculated using SAS (SAS, 1990).

### RESULTS

### Correlation of IL-1α expression in bladder carcinomas with patient survival

RNA was isolated from a total of 95 urinary bladder tumour biopsies collected during 1995–1996. After reverse transcription, samples were subjected to quantiative PCR using the TaqMan method (Heid et al, 1996). Only samples where the 18S rRNA internal standard could be adequately amplified during a predetermined number of cycles were included, since samples that contained smaller quantities of amplifiable cDNA did not yield accurate determinations of IL-1α or uPA (see Methods). The bladder carcinomas from 73 patients with a median age of 74 years, ranged from 35 to 99 year, and with a median follow up of 37 months fulfilled this requirement and were included in the analysis (60 patients for uPA). The properties of these patients are shown in Table 1. A relation was found between mortality and low differentiation of tumours, the relative risk (with 95% confidence interval) being 2.01 (0.85–4.7) for G2b+G3 versus G1+G2a tumours (Table 2; *n* = 73). Mortality was also higher for patients with invasive tumours, the relative risk being 1.93 (0.81–4.6) for muscle invasive tumours as compared with TaG1+G2a tumours (Table 2).

IL-1α mRNA expression was examined in 73 bladder tumours (Table 3). The levels of IL-1α mRNA expression did not differ significantly between G1+G2a tumours and G2b+G3 tumours (0.17 ± 0.19 versus 0.11 ± 0.18; *P* = 0.23). Furthermore, there was no difference in IL-1α mRNA expression between Ta and T2+ tumours.

In the group of patients who were alive at the end of the follow up, the level of IL-1α mRNA expression was 0.19 ± 0.22 a.u. compared to 0.05 ± 0.07 a.u. in patients who had died during the period 1995–1999. This difference was statistically significant (Student's *t*-test; *P* = 0.0004). Using the median value of IL-1α mRNA expression for the whole material (0.040 a.u.) as a cut-off,

**Table 1** Characteristics of urinary bladder carcinoma patients

| | No. of patients (*n*) | (%) | No. of patients High IL-1α [a] | No. of patients Low IL-1α [a] |
|---|---|---|---|---|
| Men | 50 | (68.5) | 27 | 23 |
| Women | 23 | (31.5) | 10 | 13 |
| Total | 73 | | 37 | 36 |
| Dead | 31 | (42.5) | 8 | 23 |
| Alive | 42 | (57.5) | 29 | 13 |
| Tumour grade | | | | |
| G1 | 2 | (2.7) | 1 | 1 |
| G2 a | 22 | (30.1) | 15 | 7 |
| G2 b | 11 | (15.1) | 7 | 4 |
| G3 | 38 | (52.1) | 14 | 24 |
| Tumour stage | | | | |
| Ta | 31 | (42.5) | 20 | 11 |
| T1 | 6 | (8.2) | 1 | 5 |
| T2+ | 36 | (49.3) | 16 | 20 |

[a]A cut-off at 0.040 a.u. was used to classify IL-1α expression as high or 'low'.

**Table 2** The relative risk associated with IL-1α expression, grade and stage of 73 tumours

| | Dead | Total | % | RR[a] (95% C.I.) | |
|---|---|---|---|---|---|
| IL-α ≥ 0.040 a.u. | 8 | 37 | 21.6 | 1.0 | |
| IL-α ≤ 0.040 a.u | 23 | 36 | 63.9 | 2.2 | (1.0–4.4) |
| Grade: | | | | | |
| G1-G2 a | 5 | 24 | 21 | 1.0 | |
| G2b-G3 | 26 | 49 | 53.1 | 2.01 | (0.85–4.7) |
| Stage: | | | | | |
| Ta G1-G2a | 5 | 22 | 22.7 | 1.0 | |
| TaG2b-G3 | 4 | 9 | 44.4 | 1.7 | (0.5–5.2) |
| T1 | 2 | 6 | 33.3 | 1.4 | (0.32–5.7) |
| T2+ | 20 | 36 | 55.6 | 1.93 | (0.81–4.6) |

[a]RR = relative risk (ratio of proportions).

**Table 3** IL-1α and uPA expression in tumours classified by grade or stage

| | All | Alive | Deceased | IL-1 α [a] (a.u) mean ± S.E.M | | uPA[a] (a.u) mean ± S.E.M | |
|---|---|---|---|---|---|---|---|
| G1 | 2 | 1 | 1 | 0.11 | 0.1 | 0.12 | 0.06 |
| G2a | 22 | 18 | 4 | 0.17 | 0.04 | 0.12 | 0.034 |
| G2b | 11 | 7 | 4 | 0.15 | 0.056 | 0.12 | 0.045 |
| G3 | 38 | 16 | 22 | 0.10 | 0.03 | 0.49 | 0.135 |
| Total | 73 | 42 | 31 | | | | |
| Ta | 31 | 22 | 9 | 0.15 | 0.03 | 0.16 | 0.06 |
| T1 | 6 | 4 | 2 | 0.05 | 0.03 | 0.3 | 0.122 |
| T2+ | 36 | 16 | 20 | 0.13 | 0.03 | 0.45 | 0.123 |
| Total | 73 | 42 | 31 | | | | |

[a]IL-1α (*n* = 73), uPA (*n* = 60). 15 of the tumours used for the evaluation of IL-1α mRNA expression could not be used for determination of uPA mRNA expression and 2 of the tumours used for the evaluation of uPA mRNA expression could not be used for the determination of the IL-1α mRNA expression.

© 2001 Cancer Research Campaign

**Table 4** The hazard ratio of mortality adjusted for age, stage and grade

| Variable | Crude[a] HR | HR adjusted for age | HR adjusted for stage | HR adjusted for grade | Hr adjusted for age, stage and grade (95% CI) |
|---|---|---|---|---|---|
| IL-1α | 4.2 | 3.5 | 3.8 | 3.6 | 3.3 (1.5–7.4) |
| uPA | 3.1 | 3.1 | 3.0 | 2.6 | 3.1 (1.2–8.1) |

[a]HR = hazard ratio.

survival rates were calculated using the Kaplan-Meier method. A decrease in overall survival time was evident for patients with low levels of IL-1α mRNA in their tumours (log rank; $P$ = 0.0002) (Figure 1A). The relative risk associated with IL-1α expression was 2.2 (1.0–4.4) for low versus high (median split) expression (Table 2). Using a Cox regression model, the hazard ratio for low versus high IL-1α expression was 4.2. After adjusting for age, stage and grade, the hazard ratio was 3.3 (Table 4).

The association between IL-1α expression and overall survival was examined in the subgroup of patients with poorly differentiated (G2b+G3) tumours (Figure 1B). Low IL-1α expression was significantly associated with a decreased overall survival time of these patients (log rank; $P$ = 0.0048). Low IL-1α expression was also associated with a decreased overall survival time of patients with invasive (T2+) tumours (log rank; $P$ = 0.02) (Figure 1C).

### Inverse relationship between IL-1α and uPA expression levels uPA mRNA expression was examined in 60 tumour biopsies. A specific pattern was seen for the relation between expression of IL-1α and uPA (Figure 2). High levels of IL-1α expression was primarily seen for low levels of uPA expression, and vice versa. uPA expression was about 4-fold higher in high grade (G3) tumours compared to G2a tumours and a similar difference was observed between T2+ and Ta tumours (Table 3). Patients with high levels of uPA mRNA in their tumours (using a medium split of 0.084 a.u.) showed a shorter over-all survival than patients with low levels of uPA mRNA (log rank $P$ = 0.032) (Figure 3). Using a Cox regression model, the hazard ratio for high versus low uPA expression was 3.1 (Table 4).

In a model including both IL-1α and uPA together with age, stage and grade, we obtained a hazard ratio of 2.9 (1.1–6.6) for IL-1α and 3.2 (1.2–8.0) for uPA.

### DISCUSSION

We found that low endogenous IL-1α mRNA expression in bladder cancer is significantly correlated to a decrease in the overall survival time of patients. Of particular interest is the finding that tumour IL-1α expression provides information with

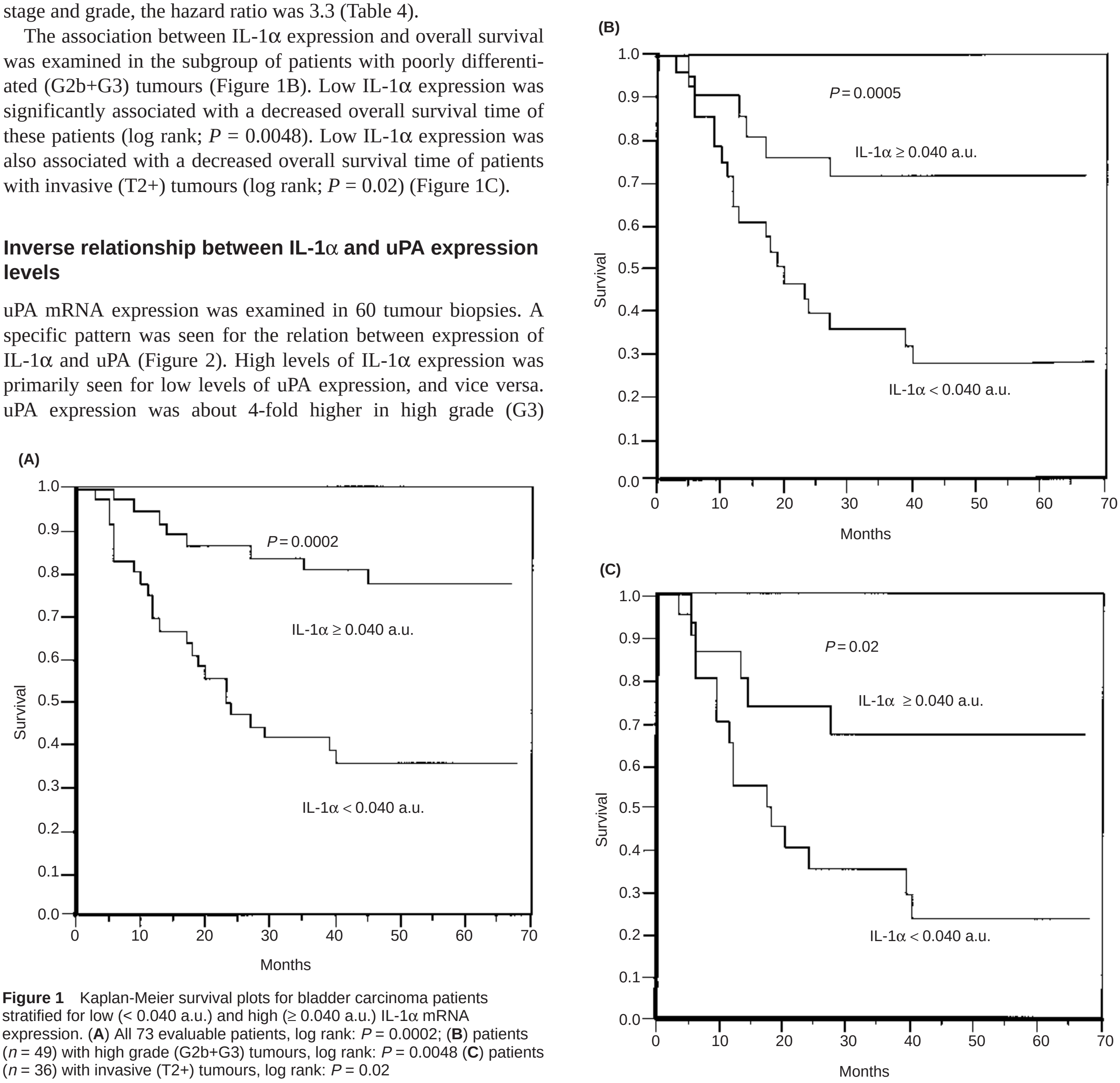

**Figure 1** Kaplan-Meier survival plots for bladder carcinoma patients stratified for low (< 0.040 a.u.) and high (≥ 0.040 a.u.) IL-1α mRNA expression. (**A**) All 73 evaluable patients, log rank: $P$ = 0.0002; (**B**) patients ($n$ = 49) with high grade (G2b+G3) tumours, log rank: $P$ = 0.0048 (**C**) patients ($n$ = 36) with invasive (T2+) tumours, log rank: $P$ = 0.02

 © 2001 Cancer Research Campaign

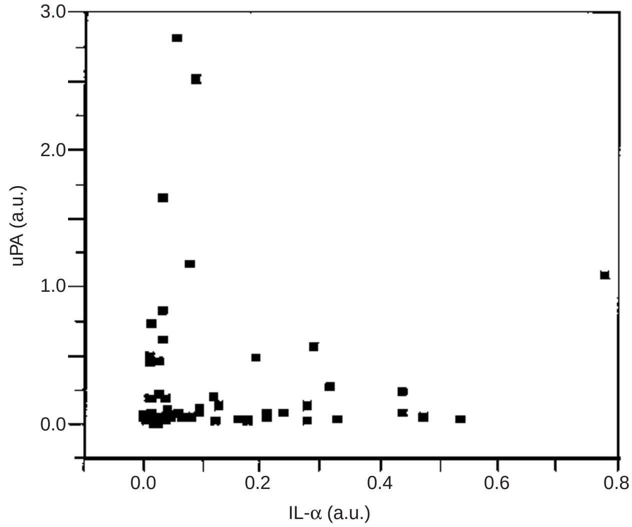

**Figure 2** Relationship between IL-1α and uPA mRNA expression in bladder carcinoma. Arbitrary units (a.u.) of mRNA expression are plotted ($n = 58$)

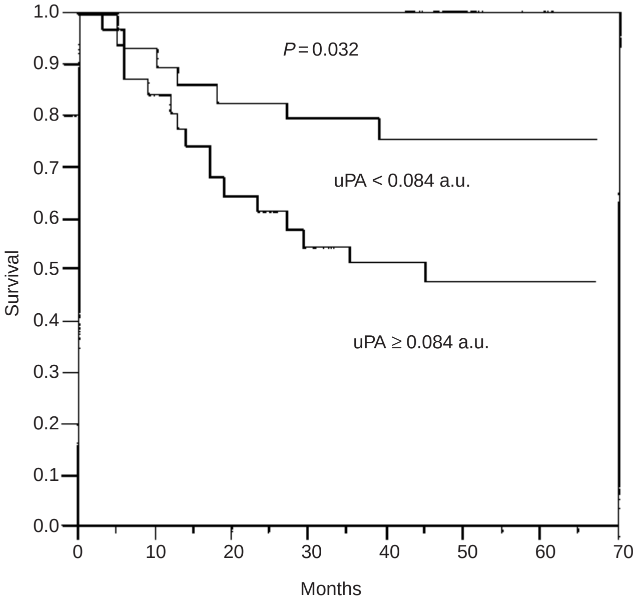

**Figure 3** Kaplan-Meier survival plots for 60 bladder carcinoma patients stratified for low (< 0.084 a.u.) and high (≥ 0.084 a.u.) uPA mRNA expression (log rank: $P = 0.032$)

regard to survival in highly malignant tumours (poorly differentiated and muscle invasive) (Figure 1B and 1C). If our results are confirmed in independent patient series, pre- treatment IL-1α expression may be clinically useful to predict the risk of progression in invasive bladder neoplasms.

The result of our study is consistent with the hypothesis that IL-1α limits the growth of bladder tumours. There are several possible, and feasibly additive, mechanisms for anti- tumoral effects by IL-1α, including inhibition of tumour cell growth (Onozaki et al, 1985), anti-angiogenic activities (Norioka et al, 1987) and upregulation of the immunological response (Dinarello, 1996). In experimental systems, forced expression of IL-1α leads to decreased tumorogenicity, and is believed to exert adjuvant-like effects increasing the immunogenicity of tumour-cell antigens (Douvdevani, 1992; Voronov et al, 1999). It has previously been suggested that the survival rate of bladder cancer patients with tumours showing inflammatory reaction is increased (Flamm, 1990). Further experiments are necessary to clarify the mechanisms of the anti-tumoral effects by IL-1α in bladder cancer, and to demonstrate which cell type secretes the cytokine.

BCG can diminish recurrence rates in superficial bladder cancer (Lamm, 1985). After incubation with BCG, monocytes from treated patients show increased IL-1α and TNF-α production. It has been suggested that the beneficial immuno- therapeutic effects of BCG in bladder cancer patients are related to its capacity to prime macrophages to increase the release of IL-1α and TNF-α (Conti et al, 1994).

uPA expression has been described as being stimulated by IL-1 (Leizer et al, 1987; Michel and Quertermous, 1989). By contrast our data indicate that uPA production primarily occurs when IL-α expression is low. Other factors, such as FGF-2 and EGF may be more important for induction of uPA expression (Visscher et al, 1995; Sieuwerts et al, 1999).

We found however a correlation between high uPA mRNA expression and short survival of bladder cancer patients. Although uPA protein levels have been reported to predict survival patients with this disease (Hasui et al, 1992), no previous study of this or any other type of tumour has demonstrated that total uPA mRNA expression also is predictive for patient outcome.

In the present series, many of the bladder cancer biopsies were small, resulting in low yields of RNA (as low as 0.4 µg). We were able to measure IL-1 expression in 73 and uPA expression in 60 of a total of 95 tumours. The reason for excluding 35 samples was their unacceptable Ct-values for the endogenous control, presumably due to RNA degradation. For the TaqMan RT-PCR method to be used in future clinical practice, these technical problems must be solved. Our results indicate that pretreatment measurements of IL-1α expression could be investigated as a possible marker for lack of invasive potential and this may reduce the number of patients undergoing unnecessary cystectomies or systemic treatments with cytostatic drugs.

### ACKNOWLEDGEMENTS

We are very grateful to Helene Rink who provided us with some of the statistical analysis. This work was supported by grants from King Gustav V Jubilee Foundation and Cancerfonden.

### REFERENCES

Bergkvist A, Ljungqvist A and Moberger G (1965) Classification of bladder tumours based on the cellular pattern. Preliminary report of a clinical-pathological study of 300 cases with a minimum follow-up of eight years. *Acta Chirurgica Scandinavica* **130**: 371–378

Bhat-Nakshatri P, Newton TR, Goulet R Jr and Nakshatri H (1998) NF-kappaB activation and interleukin 6 production in fibroblasts by estrogen receptor-negative breast cancer cell-derived interleukin 1alpha. *Proc Natl Acad Sci USA* **95**: 6971–6976

Bohle A (1999) BCG's Mechanism of action – increasing our understanding. *Eur Urol* **37(S1)**: 1–8

Brinckerhoff CE, Sirum-Connolly K, Karmilowicz MJ and Auble DT (1992) Expression of stromelysin and stromelysin-2 in rabbit and human fibroblasts. *Matrix* **1**: 165–175

Burrows FJ, Haskard DO, Hart IR, Marshall JF, Selkirk S, Poole S and Thorpe PE (1991) Influence of tumor-derived interleukin 1 on melanoma-endothelial cell interactions in vitro. *Cancer Res* **51**: 4768–4775

© 2001 Cancer Research Campaign 

Conti P, Reale M, Nicolai M, Barbacane RC, Placido FC, Iantorno R and Tenaglia R (1994) Bacillus Calmette-Guerin potentiates monocyte responses to lipopolysaccharide-induced tumor necrosis factor and interleukin-1, but not interleukin-6 in bladder cancer patients. *Cancer Immunol Immunother* **38**: 365–371

Dinarello CA (1996) Biologic basis for interleukin-1 in disease. *Blood* **87**: 2095–2147

Dinarello CA (1997) Interleukin-1. *Cytokine Growth Factor Rev* **8**: 253–265

Douvdevani A, Huleihel M, Zoller M, Segal S and Apte RN (1992) Reduced tumorigenicity of fibrosarcomas which constitutively generate IL-1 alpha either spontaneously or following IL-1 alpha gene transfer. *Int J Cancer* **51**: 822–830

Engel G, Heselmeyer K, Auer G, Bäckdahl M, Eriksson E and Linder S (1994) Correlation between stromelysin-3 mRNA level and outcome of human breast cancer. *Int J Cancer* **58**: 830–835

Epstein JI (1998) Bladder consensus conference committee, The World Health Organization/International Society of Urological Pathology consensus classification of urothelial (translational cell) neoplasm of the urinary bladder. *Am J Surg Pathol* **22**: 1435–148

Flamm J (1990) The value of tumor-associated tissue inflammatory reaction in primary superficial bladder cancer. *Urol Res* **18**: 113–117

Hall RR and Prout GR (1990) Staging of bladder cancer: is the tumor, node, metastasis system adequate? *Semin Oncol* **17**: 517–523

Hasui Y, Marutsuka K, Suzumiya J, Kitada S, Osada Y and Sumiyoshi A (1992) The content of urokinase-type plasminogen activator antigen as a prognostic factor in urinary bladder cancer. *Int J Cancer* **50**: 871–873

Hayashi O, Akashi M, Fujime M, Hanazawa K and Kitagawa R (1994) Detection of interleukin-1 activity in human bladder cancer cell lines. *J Urol* **151**: 750–753

Heid CA, Stevens J, Livak KJ and Williams PM (1996) Real time quantitative PCR. *Genome Research* **6**: 986–994

Hellsten S and Steineck G (1997) Bladder cancer in Sweden. *Med Oncol* **14**: 141–143

Janik JE, Miller LL, Longo DL, Powers GC, Urba WJ, Kopp WC, Gause BL, Curti BD, Fenton RG, Oppenheim JJ, Conlon KC, Holmlund JT, Sznol M, Sharfman WH, Steis RG, Creekmore SP, Alvord WG, Beauchamp AE and Smith JW 2nd (1996) Phase II trial of interleukin 1 alpha and indomethacin in treatment of metastatic melanoma. *J Natl Cancer Inst* **88**: 44–49

Jin L, Yuan RQ, Fuchs A, Yao Y, Joseph A, Schwall R, Schnitt SJ, Guida A, Hastings HM, Andres J, Turkel G, Polverini PJ, Goldberg ID and Rosen EM (1997) Expression of interleukin-1 beta in human breast carcinoma. *Cancer* **80**: 421–434

Kaplan EL and Meier P (1955) Nonparametric estimation from incomplete observations. *J Am Stat Assoc* **53**: 457–481

Lamm DL (1985) Bacillus Calmette-Guerin immunotherapy for bladder cancer. *J Urol* **134**: 40–47

Leizer T, Clarris BJ, Ash PE, van Damme J, Saklatvala J and Hamilton JA (1987) Interleukin-1 beta and interleukin-1 alpha stimulate the plasminogen activator activity and prostaglandin E2 levels of human synovial cells. *Arthritis Rheum* **30**: 562–566

Linder C, Bystrom P, Engel G, Auer G, Aspenblad U, Strander H and Linder S (1998) Correlation between basic fibroblast growth factor immunostaining of stromal cells and stromelysin-3 mRNA expression in human breast carcinoma. *Br J Cancer* **77**: 941–945

Liotta LA and Stetler-Stevenson WG (1993) Principles of molecular cell biology of cancer: cancer metastasis. *Canc Principl & Practice of Onc* **4**: 134–146

Michel JB and Quertermous T (1989) Modulation of mRNA levels for urinary- and tissue-type plasminogen activator and plasminogen activator inhibitors 1 and 2 in human fibroblasts by interleukin 1. *J Immunol* **143**: 890–895

Norioka K, Hara M, Kitani A, Hirose T, Hirose W, Harigai M, Suzuki K, Kawakami M, Tabata H, Kawagoe M and Nakamura H (1987) Inhibitory effect of human recombinant interleukin-1 alpha and beta on growth of human vascular endothelial cells. *Biochem Biophys Res Commun* **145**: 969–975

Okada A, Bellocq JP, Rouyer N, Chenard MP, Rio MC, Chambon P and Basset P (1995) Membrane-type matrix metalloproteinase (MT- MMP) gene is expressed in stromal cells of human colon, breast, and head and neck carcinomas. *Proc Natl Acad Sci USA* **92**: 2730–2734

Onozaki K, Matsushima K, Aggarwal BB and Oppenheim JJ (1985) Human interleukin 1 is a cytocidal factor for several tumor cell lines. *J Immunol* **135**: 3962–3968

Pyke C, Kristensen P, Ralfkiaer E, Grondahl-Hansen J, Eriksen J, Blasi F and Dano K (1991) Urokinase-type plasminogen activator is expressed in stromal cells and its receptor in cancer cells at invasive foci in human colon adenocarcinomas. *Am J Pathol* **138**: 1059–1067

Rothman KJ and Greenland S (eds) (1998) *Modern epidemiology*, 2nd ed., Lippincott-Raven: Philadelphia

SAS procedure guide, version 6. 3rd ed. (1990) SAS Institute, Cary, NC

Shipley WU, Winter KA, Kaufman DS, Lee WR, Heney NM, Tester WR, Donnelly BJ, Venner PM, Perez CA, Murray KJ, Doggett RS and True LD (1998) Phase III trial of neoadjuvant chemotherapy in patients with invasive bladder cancer treated with selective bladder preservation by combined radiation therapy and chemotherapy: initial results of Radiation Therapy Oncology Group 89–103. *J Clin Oncol* **16**: 3576–3583

Sieuwerts AM, Klijn JG, Henzen-Logmans SC and Foekens, JA (1999) Cytokine-regulated urokinase-type-plasminogen-activator (uPA) production by human breast fibroblasts in vitro. *Breast Cancer Res Treat* **55**: 9–20

Stein JP, Ginsberg DA, Grossfeld GD, Chatterjee SJ, Esrig D, Dickinson MG, Groshen S, Taylor CR, Jones PA, Skinner DG and Cote R (1998) Effect of p21WAF1/CIP1 expression on tumor progression in bladder cancer. *J Natl Cancer Inst* **90**: 1072–1079

Steineck G, Cordon-Cardo D and Scher HI (1997) Bladder and uroepithelial carcinomas. In: W Schrier and CW Gottschalk (eds), *Diseases of the Kidney*, pp. 803–822. Little Brown and Company, New York

Tetu B, Brisson J, Cote C, Brisson S, Potvin D and Roberge N (1993) Prognostic significance of cathepsin-D expression in node-positive breast carcinoma: an immunohistochemical study. *Int J Cancer* **55**: 429–435

Visscher DW, DeMattia F, Ottosen S, Sarkar FH and Crissman JD (1995) Biologic and clinical significance of basic fibroblast growth factor immunostaining in breast carcinoma. *Modern Pathology* **8**: 665–670

Voronov E, Weinstein Y, Benharroch D, Cagnano E, Ofir R, Dobkin M, White RM, Zoller M, Barak V, Segal S and Apte RN (1999) Antitumor and immunotherapeutic effects of activated invasive T lymphoma cells that display short-term interleukin 1alpha expression. *Cancer Res* **59**: 1029–1035

Wolf C, Rouyer N, Lutz Y, Adida C, Loriot M, Bellocq J-P, Chambon P and Basset P (1993) Stromelysin 3 belongs to a subgroup of proteinases expressed in breast carcinoma fibroblastic cells and possibly implicated in tumor progression. *Proc Natl Acad Sci USA* **90**: 1843–1847

Worth LL, Jaffe N, Benjamin RS, Papadopoulos NE, Patel S, Raymond AK, Jia SF, Rodriguez C, Gano J, Gianan MA and Kleinerman ES (1997) Phase II study of recombinant interleukin 1alpha and etoposide in patients with relapsed osteosarcoma. *Clin Cancer Res* **3**: 1721–1729

*© 2001 Cancer Research Campaign*
